# Applying systems biology methods to the study of human physiology in extreme environments

**DOI:** 10.1186/2046-7648-2-8

**Published:** 2013-03-22

**Authors:** Lindsay M Edwards, Ines Thiele

**Affiliations:** 1 Centre of Human & Aerospace Physiological Sciences, School of Biomedical Sciences, King’s College London, London, England, SE1 1UL, UK; 2 Center for Systems Biology, University of Iceland, Reykjavik, IS-101, Iceland

## Abstract

Systems biology is defined in this review as ‘an iterative process of computational model building and experimental model revision with the aim of understanding or simulating complex biological systems’. We propose that, in practice, systems biology rests on three pillars: computation, the *omics* disciplines and repeated experimental perturbation of the system of interest. The number of ethical and physiologically relevant perturbations that can be used in experiments on healthy humans is extremely limited and principally comprises exercise, nutrition, infusions (e.g. Intralipid), some drugs and altered environment. Thus, we argue that systems biology and environmental physiology are natural symbionts for those interested in a system-level understanding of human biology. However, despite excellent progress in high-altitude genetics and several proteomics studies, systems biology research into human adaptation to extreme environments is in its infancy. A brief description and overview of systems biology in its current guise is given, followed by a mini review of computational methods used for modelling biological systems. Special attention is given to high-altitude research, metabolic network reconstruction and constraint-based modelling.

## Review

### Introduction

The term ‘systems biology’ is suddenly everywhere. One could be forgiven for believing, therefore, that it is something new, yet this would be misleading. The application of general system theory to living organisms dates back, at least, to Ludwig von Bertalanffy's pioneering work in the 1920s and perhaps earlier still (see [1] and references therein). To appreciate exactly what systems biology is, at least in theory and philosophy, one could do worse than von Bertalanffy's own description of a system and why general system theory rose to prominence in the last century. He contrasted system theory with the analytical, reductionist approach to science that had characterised the scientific method from Francis Bacon onward, thus, “Application of the analytical procedure depends on two conditions. The first is that interactions between ‘parts’ be… weak enough to be neglected … Only under this condition, can the parts be ‘worked out,’ actually, logically and mathematically, and then be ‘put together.’ The second condition is that the relations describing the behaviour of the parts be linear” [1].

The first condition is clear enough. The second can be better expressed mathematically. If each ‘part’ of the whole can be described by a function (say *f*_1_, *f*_2_ etc.), then condition two states that the behaviour of the whole (say *w*) can be written as a linear combination of the parts, i.e. *w* = *x*_1_*f*_1_ + *x*_2_*f*_2_ + *x*_3_*f*_3_… where *x*_1_, *x*_2_… etc. are constants. For von Bertalanffy, violation of either or both of these conditions *defined* a system [1]. Few with any experience of biological research would argue that these conditions are true of living things. Life is, by its very nature, awesomely complex and chaotic. To cite von Bertalanffy one final time, ‘The then prevalent mechanistic approach just mentioned appeared to neglect or actively deny just what is essential in the phenomena of life.’

This, then, helps define the theory and philosophy of systems biology. Yet, what does it mean in practice? That is the focus and purpose of this review. To some extent, this review will be biased by the authors' own experiences of working together in this area. Thus, examples will be drawn from high-altitude research and the use of metabolic network reconstructions. Yet, these perspectives, we hope, will be broadly generalisable, and we aim to provide a basic introduction to systems biology for the non-expert. We also propose that systems biology and environmental and exercise physiology are particularly complementary. We will also briefly discuss some of the challenges that lie ahead.

### What is (and is not) systems biology?

If then it is nothing new, why is systems biology suddenly so visible? Some have implicitly argued that systems biology is a mirage, no more than a rebranding of the type of holistic thinking that some biologists and integrative physiologists have been using for decades [2]. Yet, systems biology in its current guise *is* different to these earlier disciplines. It stems from advances in technology, particularly in genome sequencing, computing and in analytical platforms such as mass spectrometry and nuclear magnetic resonance. In order to truly study a large system in its entirety, one requires the ability to model and measure it in its entirety (or at least make an effective attempt to do so). Until the advent of whole genome sequencing, this was an insurmountable experimental challenge for biologists. With the advancements in computing power, genomics, transcriptomics, proteomics, metabolomics and fluxomics, it is becoming possible to ‘profile’ and model a complete biological system or subsystem.

Yet, another common misconception is that systems biology and the so-called *omics* disciplines are one and the same. This, too, is misleading. An omics discipline is defined by its methods, as an attempt to measure every instance of a species in a specific class. Thus, proteomics is an attempt to measure every protein in a cell or tissue. Systems biology, while leveraging much of the data these experiments generate, transcends the methods. Reconstructions of cell metabolic networks not only undoubtedly leverage data from genomics, proteomics and metabolomics, but also use data from traditional enzyme assays and measures of physiological function. Indeed, a key test of any reconstruction is whether it has the capacity to recapitulate the normal physiological functions of the system of interest [3]. Another feature that distinguishes systems biology from omics disciplines is recursivity. Systems biology as defined herein (and elsewhere [4]) comprises an iterative cycle of experiment and modelling rather than a single experiment and modelling cycle. In this respect, systems biology is similar to traditional biological practice, in which statistics are used to ‘process’ experimental data before the ‘filtered’ observations are compared with a biological model, leading to progressive model revision. This has been described as the ‘model as hypothesis’ [4].

**Figure 1:**
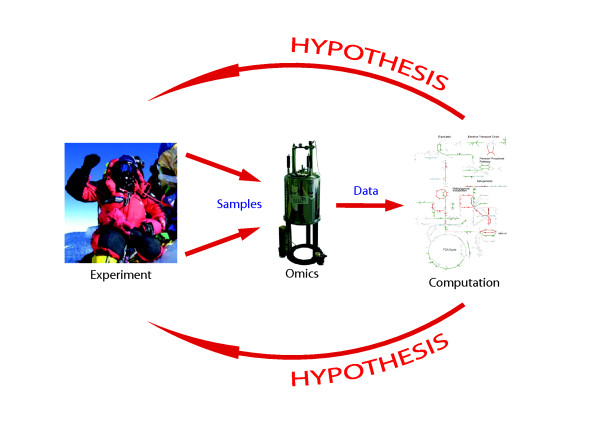
The iterative cycle of systems biology (image of climber courtesy of Caudwell Xtreme Everest).

In practice, perhaps the single feature that distinguishes systems biology from both the omics disciplines and other relatives such as integrative physiology is the central role of mathematical modelling and computer simulations—a distinction that has been overlooked by previous commentators [5]. Yet, we are not alone in emphasising the central role of computation in systems biology [6-8]. Therefore, we propose defining systems biology (for the purposes of this review) as ‘an iterative process of computational model building and experimental model revision with the aim of understanding or simulating complex biological systems’ (Figure 1). Although there has been substantial variation in how systems biology has been defined previously, we believe that our definition here is consistent with the emerging consensus [4,6,9]. Given the huge amounts of data generated by the trademark techniques of genomics, transcriptomics, proteomics and metabolomics, unaided human interpretation is utterly inadequate, and systems biology, as defined here and elsewhere, arises spontaneously. Yet, typical omics experiments (in isolation) fail this test; most involve the *de novo* building of multivariate statistical models to the data from each experiment in isolation rather than progressive model refinement (statisticians who specialise in multivariate modelling are well aware of the problems associated with this approach; instead, they recommend that such statistical models are confirmed using newly acquired data or bootstrapping procedures [10]).

There are many in the biological sciences who continue to hold that the very phrase ‘biological modelling’ is an oxymoron. To them, we argue that model building itself is an indispensible (yet often tacit) part of biological thinking. Whether the model is descriptive only (as is found in the discussion section of virtually every biological paper ever published), graphic (i.e. a figure or drawing) or fully quantitative, all models are nothing more than descriptions of biological reality that one believes to be correct. Adding numbers to a model simply makes its validity easier to assess; computers are required if the complexity of a quantitative (or even semi-quantitative) model passes beyond a certain point.

### Approaches to modelling biological systems

If mathematical modelling and computer simulations are the distinguishing features of systems biology, which methods are currently used? This review is too limited a forum for examining such a broad topic in any depth; fortunately, many excellent books and reviews already exist (see [4,11-13]). Nevertheless, a brief introduction follows, preceding a more in-depth description of one particular method: genome-scale biochemical modelling.

**Figure 2:**
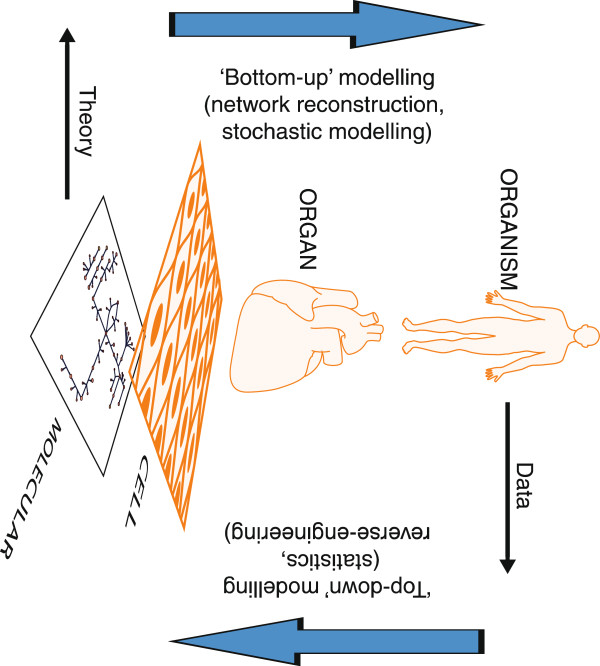
‘Top-down’ vs. ‘bottom-up’ approaches to modelling biological systems viewed in the context of biological hierarchies.

Approaches to modelling biological systems can broadly be divided into two, often described as ‘top-down’ and ‘bottom-up’ (Figure 2). Top-down methods start with data and fit models to them. Traditional statistics is therefore a top-down method, as are machine learning, pattern recognition and (broadly) bioinformatics. These methods can discern meaningful biological relationships and sometimes even quite complex networks; in such cases, they are often referred to as ‘reverse-engineering’. For example, Algorithm for the Reconstruction of Accurate Cellular Networks (ARACNE) uses statistical methods to reconstruct transcriptional networks by extracting correlated transcripts across a series of biological perturbations [14]. ARACNE successfully predicted 11 out of 12 targets of the *MYC* transcription factor, based on perturbation experiments, which were subsequently experimentally validated [15]. Of the multivariate statistical methods currently in fashion, perhaps the most popular is principal component analysis (PCA) and its relatives (such as orthogonal projection onto latent spaces). These techniques are regularly used to process omics data, especially metabolomics.

In contrast to top-down methods, such as ARACNE and PCA, are bottom-up methods that attempt to build models based on existing or acquired knowledge of network behaviour and connectedness (or topology). These include traditional kinetic models (based on a system of differential equations, *cf*. [16]) and stochastic methods [17]. However, both these approaches (kinetic and stochastic) are presently limited to relatively small systems. An exciting new approach to bottom-up modelling is the reconstruction of biochemical networks combined with a suite of modelling tools that fall under the heading of constraint-based modelling [18]. The reconstruction process is well established for metabolism and has been applied to a growing number of organisms, including mouse [19] and human [20]. The same approach can also be applied for other cellular functions, such as signalling [21,22] and macromolecular synthesis [23]. The reconstruction process has been reviewed by numerous groups [24-26], and more recently, a standard operating procedure has been formulated that describes the necessary steps in great detail [3].

Lastly, it should be noted that the distinction between top-down and bottom-up modelling is not one of biological hierarchical level. For example, one might use metabolomics data acquired from cells and build a statistical model from it—this would be top-down modelling of a system. Equally, one could build a model of signal transduction (from organ to organ) in the blood using theories of flow and diffusion and anatomical measurements. This would be a bottom-up model.

### Metabolic network reconstruction

We will briefly introduce the general approach to reconstructing metabolic networks; a more detailed description can be found elsewhere [3]. First, a draft reconstruction is generated based on an annotated genome (for example, from NCBI). Biochemical databases such as Kyoto Encyclopedia Of Genes And Genomes (KEGG) [27] and Braunschweig Enzyme Database [28] are used to link genes to function and thus metabolic reactions. However, the resulting draft reconstruction will be incomplete and will contain numerous missing or wrong annotations. The refinement and expansion of the draft reconstruction is performed through manual curation and extensive use of biochemical literature specific for the target organism. Particular attention needs to be paid to substrate and coenzyme specificity, which can differ between organisms and may require additional biochemical data. As a third step, the conversion of the manually curated metabolic reconstruction into a mathematical model follows, which includes the addition of physico-chemical and physiological balances and bounds (or constraints). Balances in biochemical networks can be, for example, mass and energy conservation, and the majority of modelling applications of metabolic models assume the system to be in quasi-steady state. Bounds on metabolic reactions can include maximal reaction rates based on the catalysing enzyme's properties (or measured *in vivo* metabolite uptake rates, for example VO_2_max) and thermodynamic information (e.g. reaction directionalities [29,30]). Network debugging and evaluation comprise the fourth stage and ensure that the metabolic model has similar phenotypic properties as the target organism. This includes evaluation of dead-end metabolites (metabolites that are only either produced or consumed in the network, but not both) to identify whether they can be connected to the remaining network by adding one or more reactions to the reconstruction. Also, the model's capability to produce its own biomass is evaluated. This process may lead to the identification of yet more network gaps. This stage also includes further quality tests; these will depend on the properties of the target organism as well as the availability of experimental data (for example, phenotyping data, knock-out data etc.). The fifth and final stage is undoubtedly the most exciting: study of the biological system of interest. Network reconstructions have been put to numerous uses over the last decade or so, including biological discovery [31], metabolic engineering [32,33], prediction of the outcome of adaptive evolution [34,35], network topology [36,37] and the assessment of phenotypic behaviour [38-41]. Some of these applications have been summarised in excellent recent reviews [42-44].

### The human metabolic reconstruction

The genome-scale metabolic reconstruction process described above was applied to human metabolism, and the subsequent reconstruction, published in 2007, was named Recon 1 [20]. Recon 1 accounts for the functions of 1,496 open reading frames, 2,766 metabolites and 3,311 reactions distributed over eight cellular compartments (the cytoplasm, mitochondria, nucleus, endoplasmic reticulum, Golgi apparatus, lysosome, peroxisome and the extracellular environment). This first comprehensive, genome-scale human metabolic reconstruction captures most of the known central metabolic pathways occurring in any human cell. This reconstruction has been employed for numerous biomedical studies (reviewed in [43]). Moreover, the generic human metabolic reconstruction serves as a starting point for tissue- and cell-type specific reconstructions, many of which are generated using *omics* data (e.g. transcriptomic and proteomic data) as well as some manual curation. These reconstructions include macrophages [45], hepatocytes [46], myocytes [47] and adipocytes [47]. Recently, a core cancer cell network has also been compiled by mapping the NCI-60 cancer cell line transcriptomic data onto Recon 1 [48].

The next pressing challenge is the need to generate multi-network and ‘trans-hierarchical’ (multi-scale) models. Examples of these are recent models that describe the metabolism of two human cell (or tissue) types and their interactions [47,49]. In these cases, the model comprises genome-scale metabolic networks at one level and a most basic two-node model of cell-cell interaction at the other. The complexity of even these simple models highlights the awesome task ahead if we wish to model the interactions between multiple cell types, tissues and organs, even before we have considered the physical complications of anatomy and physiology. Other important multi-system models are those attempting to reconcile metabolism with, for example, transcriptional control (*cf*. [50-52]) and those that combine gross models of physiology with detailed models of enzyme kinetics [53]. Again, an excellent recent review has addressed the challenges associated with multi-scale modelling [13].

Two final notes on bottom-up reconstructions: first, although these models are termed bottom-up, they are, inevitably, middle-up. This is because they rest on an arbitrarily defined lowest level; one could almost always model from a lower level (say atomic or subatomic). This, however, raises a serious philosophical point—is it possible to predict complex biological functions from the very lowest hierarchical levels or are we prevented from ever doing this (perhaps by a form of Gödel's completeness theorem as suggested previously [4])? Second, the level of detail (sometimes called fine- or coarse-graining) in a model is very important. Although it seems as though increasing detail would always be desirable, it may not be [6]. Parameter fitting (i.e. estimating parameters from *in vivo* data) is hazardous and can lead to mistaken confidence, especially where an unknown and unmeasured molecule (or other influence) may be acting simultaneously on multiple points in a system (as may very often be the case [7]). Another practical constraint on the degree of detail in a bottom-up model is computing power; there is no point having a model that includes single molecule dynamics if it is completely unusable.

### Systems biology at work—filling gaps in human metabolism

Figure 1 shows the iterative cycle of systems biology: model building and computation generate hypotheses that are tested experimentally, leading to further model refinement. For example, a common and important question when reconstructing metabolic networks is as follows: is the reconstruction complete? Given that metabolic network reconstructions leverage all the currently available data regarding human metabolism, this question is equivalent to asking whether our knowledge of human metabolism is complete, yet in a completely thorough and systematised way. One can identify missing reactions in a network reconstruction by comparing model simulations with experimental data [31]. This method is generally called gap filling, and numerous computational algorithms have been published [31,54,55]. Similarly, metabolomics data from cells, tissues and biofluids [56-58] could be used to identify missing knowledge in human metabolism; the presence of a metabolite in a biofluid, which is absent from the reconstruction, indicates a knowledge gap. There are a number of different computational approaches [31,55] that could be used in these cases to identify candidate missing reactions and corresponding genes [59,60]. These computational methods ‘borrow’ one or more reactions, known to be present in other species, from a universal reaction database (for example, the KEGG ligand database [27]) and add them to the metabolic model, thus potentially filling the gap. If no existing experimental support can be found in the literature, then the predicted missing genes and reactions are hypotheses that require experimental validation.

### Systems biology and extreme physiology research

How can these methods help the extreme physiology researcher (and what use are data on humans in extreme environments to the systems biologist)? Central to the practice of systems biology, at least in cells, is the concept of perturbing a biological system [6]. Thus, one might very reasonably argue that the three pillars of systems biology are (1) the ability to measure all the variables of interest (omics), (2) a conceptual framework within which to understand the data (a model) and (3) a way of perturbing the system under interrogation (the experiment). However, the list of methods available to perturb the biological homeostasis of a healthy human is relatively short and comprises exercise, drugs (subject to ethical constraints), dietary manipulation, infusions (for example, lipid emulsions such as Intralipid [58]) and environmental challenges including extreme temperature and hypoxia. Therefore, extreme environments represent one of only a handful of techniques to perturb healthy human biology in a systems biology experiment. As we stated at the outset, we believe therefore that environmental physiology and systems biology are natural symbionts. The usefulness of exercise in this role has, to a limited extent, already been recognised by a small number of systems biology researchers [61,62]. For those whose research focus is environmental adaptation, they will be well aware that the patterns of human cell, organ and physiological adaptation to extreme environments are astonishingly complex. Reductionist methods have led to a number of ‘paradoxes’ (for example, the lactate paradox in hypoxia). As suggested previously [63], resolving these paradoxes may require systems biology methods. For example, computational modelling suggests that molecular overcrowding in cells may be an important, yet overlooked, factor when attempting to explain limits to cell oxidative metabolism (and hence lactate production under various conditions) [61]. Thus, collaborations between environmental physiologists and systems biologists (with an interest in human physiology) would appear to make good sense for both parties.

Although there is currently a shortage of systems biology studies (as defined here and elsewhere [4,8]) on human environmental physiology, excellent work has been conducted in the areas of high-altitude genetics and proteomics. Several studies have shown convincingly that human populations at high altitude have experienced a degree of genetic divergence. For example, two recent studies showed that Tibetans, whose ancestors have resided at high altitude for over 10,000 years, have acquired and maintained novel mutations in the gene encoding the oxygen-sensing hypoxia-inducible factor (HIF) molecule [64,65]. Two-dimensional gel electrophoresis-based proteomics, conducted on skeletal muscle biopsies acquired from subjects exposed for 1 week at 4,500 m [66], showed a number of proteins (related to iron transport and oxidative metabolism) whose abundance was significantly different in experienced climbers after exposure to extreme high altitude. Other investigators have studied the human urinary peptidome [67] and plasma proteome [68] in response to altitude exposure, in the latter case, with particular attention to identifying biomarkers of high-altitude pulmonary oedema. These and similar studies will provide the building blocks for a concerted systems biology effort to model and understand the human physiological response to high altitude. What is required now is a computational framework within which these disparate data can be unified and examined together, most probably a network reconstruction such as that outlined above [69]. There is also a substantial literature on experimental hypoxia (and related issues such as HIF signalling) in humans and animals, including genuine systems biology research, much of which would be relevant to those with an interest in high-altitude acclimatization (*cf*. [70-77]).

### Building bridges between disciplines

Finally, here is a word regarding the challenges ahead. There is increasing interest, driven to some extent by a systems biology agenda within the major funding agencies, in building collaborations between scientists from the life scientists and their colleagues from the physical sciences (including physicists, computer scientists, chemists and mathematicians). Thus, the authors' own collaboration, between a systems biologist/bioengineer and a human physiologist, will become increasingly common. Yet, simply putting physical and life scientists ‘in the same room’ is not enough. Life scientists with poor mathematics will struggle to grasp much of what is possible, while physical scientists with little experience or knowledge of biology will fail to instinctively see both new applications and potential limitations. A perhaps neglected aspect of this interaction is that a lack of knowledge of each others' disciplines *limits the scope of the conversation.*

There is also a recognition that a new breed of trans-disciplinary scientists will be needed. At present, the focus is on retraining physical scientists and mathematicians in life sciences, with the tacit assumption that this is an easier task than retraining life scientists in mathematics and computation. This, we believe, is a hazardous course of action. Years of experience in any field is never wasted, and life scientists bring with them an innate understanding of the ‘logic of life’ that is impossible to gain in a few short weeks (or even years). We are not alone in this viewpoint; to paraphrase Ideker et al., ‘cross-disciplinary scientists’ contributions will be proportional to their understanding of *biology*[6]. Thus, physiologists and life scientists must be prepared to rise to the challenge, by expanding their knowledge of computation and (especially) mathematics to a level that will allow them to be productive systems biologists and to engage with scientists from other areas in an informed and productive manner. No longer can biology be considered a science for those who ‘cannot do maths’.

## Conclusions

Systems biology is everywhere, yet true applications of systems biology in human physiological research are surprisingly rare. This review has attempted to provide a brief overview of systems biology for the non-expert, while also attempting to describe some ways in which these new approaches can be used to further our understanding of how humans respond to extreme environments. Indeed, we argue that environmental physiology and systems biology are natural bedfellows. Environmental and exercise challenges provide a unique platform for the study of human physiology from a systems perspective, by allowing scientists to challenge homeostasis in a manner that is both ethical and evolutionarily appropriate (in other words, by challenging human physiology with challenges that it has evolved to withstand). Ultimately, the hope is that the relationship between physiology and systems biology will develop and grow, leading us to a more mature and profound understanding of healthy human biology.

## Abbreviations

ARACNE: Algorithm for the Reconstruction of Accurate Cellular Networks; HIF: Hypoxia-inducible factor; KEGG: Kyoto Encyclopedia of Genes and Genomes; PCA: Principal components analysis; Recon 1: Human metabolic network reconstruction v1; VO2max: Maximal oxygen uptake.

## Competing interests

The authors declare that they have no competing interests.
